# Double-sheeted silicone plate reconstruction for inferomedial orbital wall fractures: a retrospective single-center case series with a historical reference cohort

**DOI:** 10.1016/j.bjorl.2026.101886

**Published:** 2026-09-10

**Authors:** Kosuke Takabayashi, Yohei Maeda, Nobuya Kataoka

**Affiliations:** a Japanese Red Cross Asahikawa Hospital, Department of Otorhinolaryngology, Asahikawa City, Hokkaido, Japan; b Sapporo Medical University School of Medicine, Department of Otorhinolaryngology, Sapporo City, Hokkaido, Japan; c Japan Community Health Care Organization Osaka Hospital, Department of Otorhinolaryngology, Osaka City, Osaka, Japan; d Osaka University Graduate School of Medicine, Department of Otorhinolaryngology, Head and Neck Surgery, Suita City, Osaka, Japan; e Japanese Red Cross Asahikawa Hospital, Department of Ophthalmology, Asahikawa City, Hokkaido, Japan

**Keywords:** Transnasal approach, Transorbital approach, Silicone sheet, Enophthalmos, Ocular motility

## Abstract

**Objective:**

To evaluate postoperative enophthalmos and ocular motility following Double Silicone Procedure (DSP), contextualized against an earlier Silicone Balloon Fixation (SBF) cohort from a time-separated single-center experience.

**Methods:**

We conducted a single-center retrospective case series (April 2000 – December 2024) across a technique transition (SBF before April 2014; DSP thereafter). Primary outcomes were patient-reported enophthalmos (graded 0–4) and postoperative ocular motility (HAR%). Between-era differences were descriptively analyzed using Hodges-Lehmann estimates with 95% Confidence Intervals (95% CIs).

**Results:**

Thirty patients met the inclusion criteria (DSP, n = 10; SBF, n = 20). The median postoperative enophthalmos score was 0 (Interquartile Range [IQR] 0–0) in the DSP group (n = 10) and 1 (IQR 0–1) in the SBF group (n = 13) (Hodges-Lehmann Δ = -1.0; 95% CI -1.0 to 0.0; p = 0.008). Among DSP patients with postoperative Hertel exophthalmometry measurements (n = 8), no patient exhibited clinically perceptible enophthalmos (>2 mm); the median Hertel exophthalmometry difference was -1.0 mm (IQR -1.0 to 0.0). Postoperative HAR% was 92.1% (IQR 86.0–100.0) after DSP and 88.4% (IQR 80.4–97.0) after SBF (p = 0.397). No optic nerve injuries, infections, or implant-related complications were observed in either group.

**Conclusion:**

In this single-center experience across a surgical technique transition, DSP demonstrated favorable postoperative outcomes, with reduced enophthalmos and preserved ocular motility. Although earlier cases served as a historical reference, these exploratory findings support DSP as a safe and practical option for complex inferomedial orbital wall fractures.

**Level of evidence:**

4.

## Highlights

• DSP reduced postoperative enophthalmos compared with SBF.
• DSP achieved non-inferior ocular motility recovery.
• Silicone sheets are inexpensive, contoured, and easily removable.
• No optic nerve injuries or implant-related complications occurred.
• DSP is a safe, effective option for complex orbital wall fractures.

## Introduction

Management of orbital blowout fractures ranges from observation to surgical repair, with treatment decisions commonly based on persistent diplopia, ocular motility restriction with suspected muscle entrapment, and clinically significant or progressive enophthalmos.^1^^,^^2^ A variety of approaches and reconstructive materials have been described, including transconjunctival and transcaruncular access, endoscopic transnasal or combined techniques, and the use of autologous grafts or alloplastic implants.^2^^,^^3^ These technical choices become more critical when fractures extend to the inferomedial region, where accurate restoration of orbital contour is particularly challenging.^1^, ^2^, ^3^

Orbital inferomedial wall fractures often involve extensive defects, and because the orbit is a complex three-dimensional structure, the fracture configuration is frequently complicated. Therefore, these fractures have been reported to carry a higher risk of residual enophthalmos^4^^,^^5^ and persistent ocular motility disturbances.^5^^,^^6^ We have performed a procedure for inferomedial wall fractures involving orbital reduction and stabilization with a silicone sheet and balloon, referred to in this study as Silicone Balloon Fixation (SBF). Although this technique achieves improvement in ocular motility, it has become evident that approximately 25% of patients report subjective enophthalmos postoperatively.^7^ To address postoperative enophthalmos, we developed a novel orbital reconstruction technique known as the Double Silicone Procedure (DSP).^8^

The DSP is a reconstruction technique that employs two silicone sheets, which can be easily shaped to match the complex orbital contour.^8^^,^^9^ This procedure reduces the risk of ocular motility disturbances and optic nerve injury that may occur with the use of oversized or irregularly shaped rigid plates, which can compress the extraocular muscles^10^ or optic nerve.^11^ Furthermore, to address potential late complications associated with long-term silicone implantation, such as migration, infection, exposure, or cyst formation,^12^, ^13^, ^14^ the silicone sheets can be removed transnasally in an outpatient setting, eliminating the need for an additional surgical procedure. Moreover, a collaborative approach involving both a transnasal endoscopic and a transorbital surgeon enhances operatability and improves intraoperative safety.^15^, ^16^, ^17^

To our knowledge, the DSP is the first reported technique to leverage the contourability of silicone sheets while enabling straightforward transnasal removal, potentially reducing late implant-related complications. Accordingly, we aimed to report postoperative outcomes following DSP and to explore between-era differences by comparing these results with earlier SBF cases used as a historical reference, while acknowledging the exploratory nature of this time-separated retrospective dataset.

## Methods

### Study design and setting

We conducted a retrospective review of consecutive cases at the Department of Otorhinolaryngology, Japan Community Health Care Organization Osaka Hospital, a tertiary referral center in Osaka, Japan. Earlier cases served as a historical reference cohort. Data were collected between April 2000 and December 2024.

### Ethical statement

Surgical treatment was performed after written informed consent was obtained from all patients. The study protocol was approved by the Ethics Committee of Japanese Red Cross Asahikawa Hospital (approval number 201818-2).

### Participants

This study included consecutive patients who underwent surgical repair for pure inferomedial orbital Blowout Fractures (BOFs) during the study period. In this study, “pure BOF” was defined as an isolated orbital wall blowout fracture without associated zygomaticomaxillary complex or other facial fractures. Inclusion criteria were: (1) An inferomedial-type pure BOF confirmed on Computed Tomography (CT), and (2) Surgical reconstruction performed using either SBF or the DSP. Exclusion criteria were: (1) Insufficient data for the prespecified analyses, including missing CT imaging and/or outcome measures (Hess Area Ratio [HAR%] for ocular motility and/or enophthalmos score); (2) Use of reconstructive techniques other than SBF or DSP; (3) Loss to follow-up before postoperative findings reached clinical stability; (4) Preexisting strabismus or other conditions precluding accurate ocular motility assessment; or (5) Urgent cases requiring immediate surgery for extraocular muscle entrapment (e.g., trapdoor/linear fractures, including “white-eyed” BOF). Surgical procedures performed through March 2014 used SBF, whereas those conducted from April 2014 onward employed DSP.

### Surgical indications and patient selection

Given that not all orbital BOFs require operative intervention, patients were initially managed with observation and serial ophthalmologic assessments, including the Hess screen test. Surgery was offered when spontaneous recovery of ocular motility reached a plateau, with persistent objective and subjective motility disturbance, in conjunction with patient preference. Surgery was also offered when clinically apparent enophthalmos became evident after swelling subsided and the patient requested corrective treatment.

### Surgical techniques

#### Double Silicone Procedure^8^

**Fig. 1:**
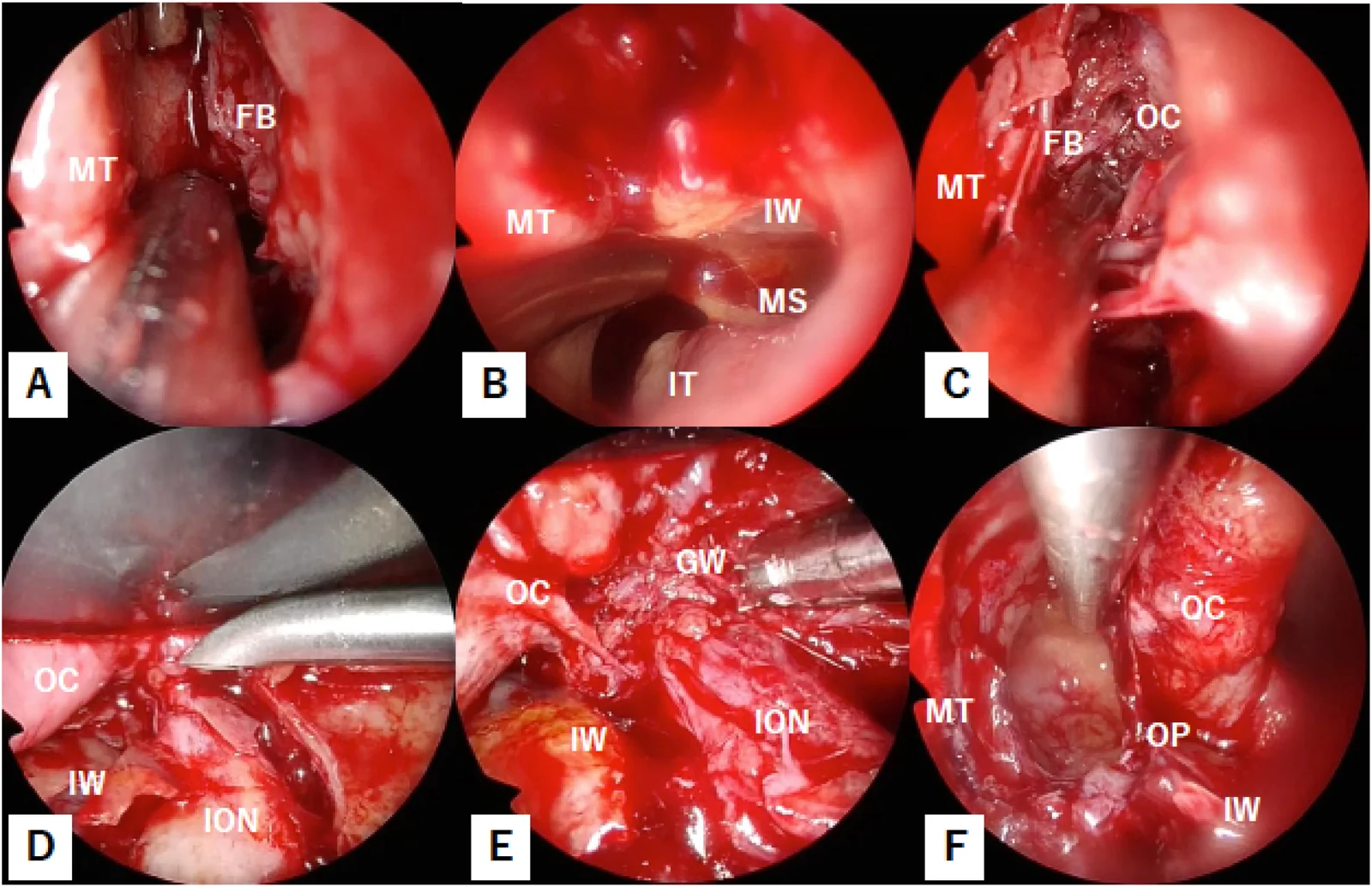
Stepwise view of the Double Silicone Procedure (DSP). Endoscopic examination through the left nasal cavity demonstrates removal of the uncinate process to expose the medial orbital wall fracture (A); resection of the maxillary sinus fontanelle subsequently opens the maxillary sinus (B); fractured bony fragments of the medial orbital wall are dissected from the orbital contents and removed (C); within the left orbit, the orbital contents are elevated from the fractured orbital floor, identifying the infraorbital nerve (D); tracing the superior margin of this nerve posteriorly identifies the inferior margin of the greater wing of the sphenoid bone (E); an endoscopic left-nasal view shows that lateralization of the orbital contents by the trans-orbital surgeon creates ample working space within the ethmoid sinus (F). FB, Fractured Bone; GW, Greater Wing of the sphenoid bone; ION, Infraorbital Nerve; IT, Inferior Turbinate; IW, Inferior Wall of the orbit; MT, Middle Turbinate; OC, Orbital Contents; OP, Orbital Process of palatine bone.

**Fig. 2:**
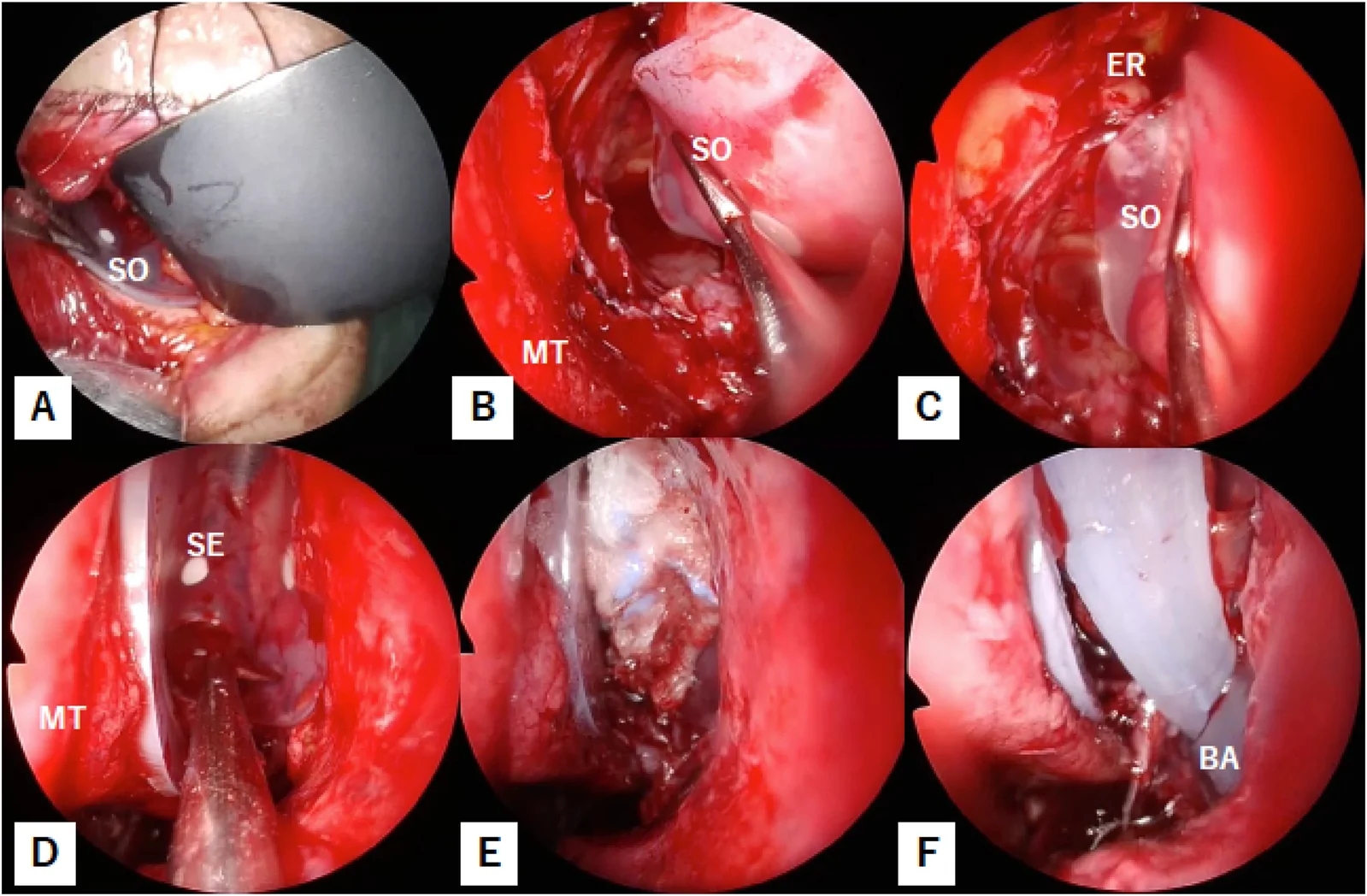
Stepwise view of the DSP, continued from Fig. 1. The first silicone sheet was placed via a transorbital route by the transorbital surgeon (A); its position was adjusted along the medial orbital wall by the transnasal surgeon (B–C) A second silicone sheet, formed into an inverted U-shape, was inserted into the ethmoid sinus and fixed with gauze soaked with ointment to reinforce the reconstructed medial wall by the transnasal surgeon (D–E); Finally, a balloon catheter was inserted within the opened maxillary sinus by the transnasal surgeon (F) to restore the orbital floor. BA, Balloon; ER, Ethmoid Roof; MT, Middle Turbinate; SE, Silicone sheet in the Ethmoid sinus; SO, Silicone sheet in Orbit.

The procedure was performed by two surgeons, one using a transnasal endoscopic approach and the other using a transorbital approach via a subciliary incision. The transnasal surgeon performed a maxillary antrostomy (Fig. 1A‒B), opened the ethmoid sinus, and removed all fractured bone fragments from the medial orbital wall (Fig. 1C). Meanwhile, the transorbital surgeon reduced the herniated orbital content while preserving the orbital floor. During this step, the transorbital approach followed the principles of the Three Landmarks Procedure by identifying the infraorbital nerve (Fig. 1D), the inferior margin of the greater wing of the sphenoid bone (Fig. 1E), and the orbital process of the palatine bone to ensure reproducible and safe dissection^15^^,^^18^ (Fig. 1F). While the transnasal surgeon performs the operation, the transorbital surgeon provides lateral retraction of the orbital contents to maintain a clear surgical field and enhance safety (Fig. 1F). In cases with severe orbital floor fractures, the transnasal surgeon may also assist with manipulation of the maxillary sinus using the prelacrimal approach.^8^ The first silicone sheet (1 mm-thick, Eyeball Restraint Insert; Koken, Tokyo, Japan) was inserted from the orbital floor by the transorbital surgeon (Fig. 2A) and adjusted along the medial orbital wall by the transnasal surgeon (Fig. 2B‒C), allowing precise placement along the contour of the orbit. A second silicone sheet was then positioned in an inverted U-shape in the opened ethmoid sinus (Fig. 2D) and fixed in place by packing with gauze soaked in ointment (Fig. 2E). Finally, a balloon was placed in the maxillary sinus to elevate and reconstruct the fractured orbital floor in its original position (Fig. 2F).

**Fig. 3:**
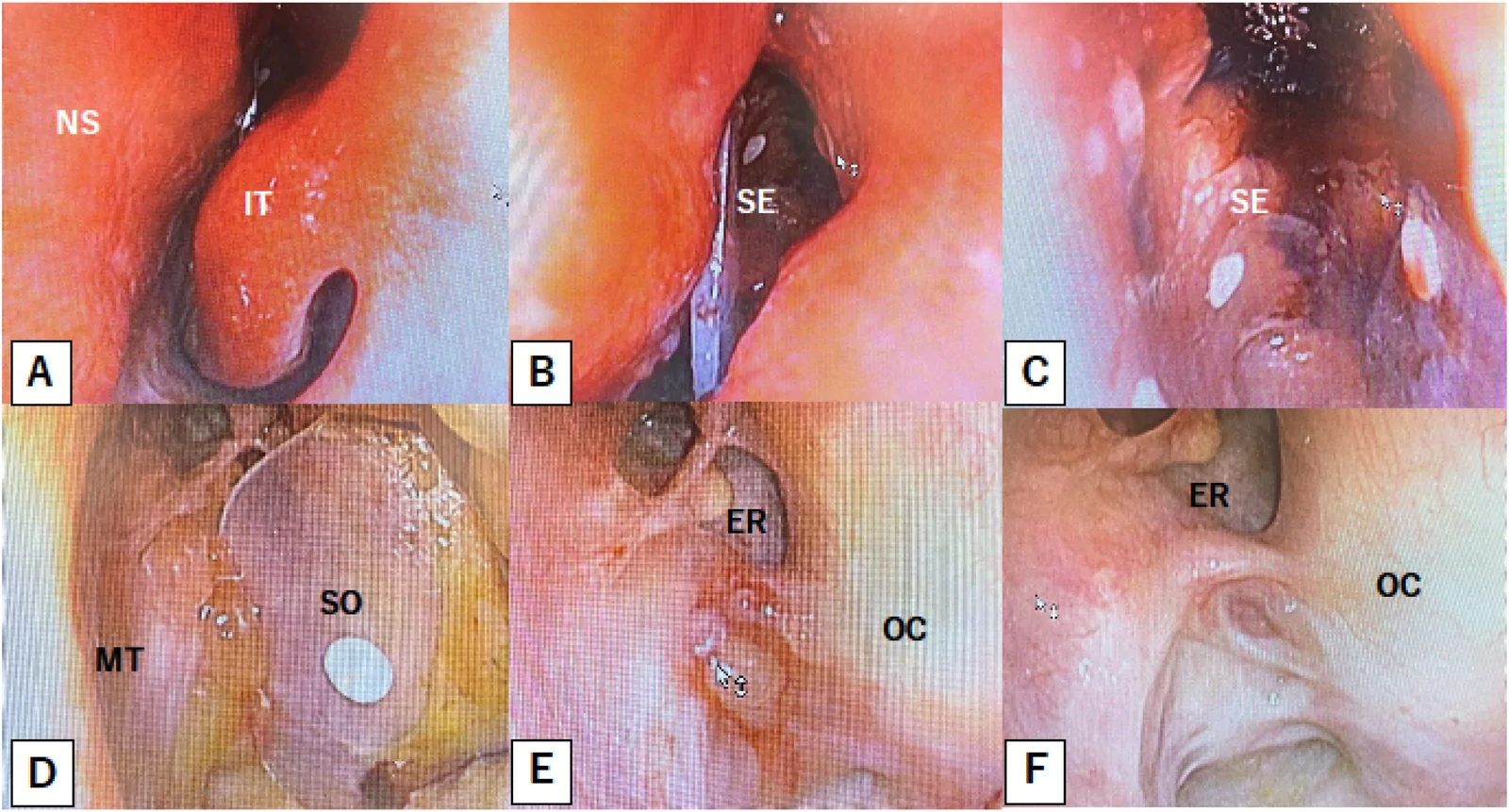
Endoscopic view of removal of the silicone sheets. An endoscopic image of the left nasal cavity is presented (A) Following the withdrawal of the gauze soaked with ointment, a silicone sheet shaped as an inverted U-configuration is visualized within the ethmoid sinus (B–C) The subsequent removal of this silicone sheet exposes a silicone sheet placed along the orbit at the medial orbital wall (D) Removal of the sheet demonstrates preservation of the medial wall by a fibrous membrane (E) The reconstructed medial orbital wall is kept one year after surgery (F). ER, Ethmoid Roof; IT, Inferior Turbinate; MT, Middle Turbinate; OC, Orbital Contents; SE, Silicone sheet in the Ethmoid sinus; SO, Silicone sheet in the Orbit.

**Fig. 4:**
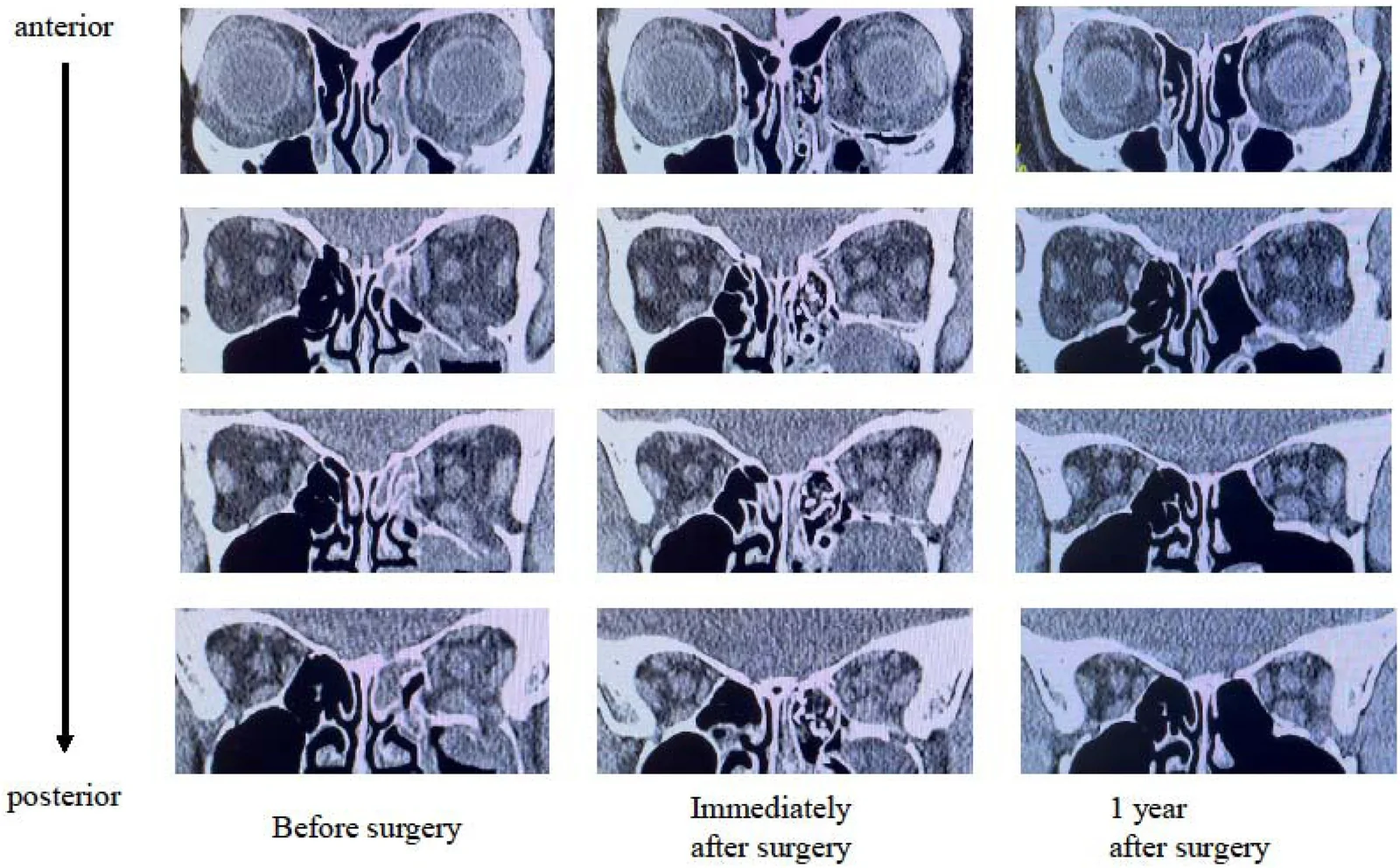
Computed Tomography (CT) images of the DSP before, immediately after surgery, and 1-year after surgery. Coronal CT images are arranged in three columns representing the preoperative (left), immediate postoperative (middle), and 1-year postoperative (right) time points. At each level, both orbits are shown on the same slice to allow comparison of vertical orbital position. Images are ordered from anterior (top row) to posterior (bottom row). Following DSP, the bony orbital floor was reconstructed, and the medial wall contour was maintained by a fibrous capsule formed along the orbital medial wall.

In this reconstruction approach, the fractured orbital floor is anatomically restored and preserved, and the bone fragments of the medial wall are completely removed. This configuration ensures that the bony orbital floor provides sufficient structural support, even after the silicone sheet is removed. On the medial side, the absence of bone allowed the silicone sheet to be retrieved from within the orbit along its contours. Fibrous tissue that formed around the silicone during postoperative healing helped maintain the orbital shape (Fig. 3). The 1-year postoperative CT image showed that the reconstructed orbital contour was well-preserved, demonstrating long-term structural stability (Fig. 4).

#### Silicone Balloon Fixation

The procedure was performed by a single surgeon using a transnasal endoscopic approach. SBF involves the complete removal of all fractured bone fragments to prevent ocular motility disturbances caused by the entrapment or adhesion of orbital contents. Orbital fixation was achieved by placing a balloon in the maxillary sinus to support the orbital floor and positioning an inverted U-shaped silicone sheet within the ethmoid sinus to stabilize the medial orbital wall.

#### Postoperative care

In both the DSP and SBF groups, the balloon was removed 7–10 days after surgery, and the silicone sheet was removed approximately 1-month postoperatively in the outpatient clinic under endoscopic transnasal guidance, without the need for return to the operating room or general anesthesia. This transnasal removal technique has been described previously, including endoscopic documentation of the removal process.^8^

#### Variables and outcome

The primary outcome measure was postoperative enophthalmos. Patients were asked to self-report their perception of enophthalmos using a five-point scale ranging from 0 to 4, where 0 indicated no subjective awareness of enophthalmos and 4 represented the highest level of perceived enophthalmos. In addition, in the DSP group, enophthalmos was quantitatively assessed using a Hertel exophthalmometer. The secondary outcome was Postoperative Ocular Motility (POM). Ocular movement was assessed using the HAR% based on the results of the Hess screen test.^19^ Preoperative HAR% was defined as the value measured after confirming spontaneous recovery of ocular motility, once further improvement had stabilized. Postoperative HAR% was defined as the value obtained after ocular motility had stabilized, either upon reaching a healed state or when no further improvement was observed. Enophthalmos scores and Hertel exophthalmometry measurements were obtained from patients whose symptoms had stabilized at least six months after surgery.

#### Data sources

All clinical data were obtained from medical records, and CT images were retrieved from the hospital’s Picture Archiving and Communication System.

#### Bias

Given the retrospective design and use of a historical reference cohort, the possibility of selection bias cannot be entirely excluded.

### Statistical methods

Continuous data are presented as medians (Interquartile Range [IQR]). Categorical variables are counts (%). Statistical analyses were performed using the free software R (version 4.4.3), with the significance level set at p < 0.05. The Mann–Whitney *U*-test and Fisher’s exact test were used for group comparisons. Between-group differences (Hodges–Lehmann estimates) and their 95% Confidence Intervals (95% CIs) were calculated using the Hodges–Lehmann method based on the Wilcoxon rank-sum test. We planned to perform a non-inferiority analysis for postoperative HAR%. Prior work shows diplopia disappears once HAR% reaches 85%,^19^ so we allowed DSP to fall at most three percentage points (pp) below SBF’s median, which is approximately 88%. With a one-sided test (α = 0.025), DSP is judged non-inferior if the lower end of its 95% CI sits above –3 pp. Because this was a retrospective study, we could not set the sample size beforehand. A preliminary calculation assuming a standard deviation of 10 pp suggests that approximately 88 patients per group would provide 80% power under these criteria.

## Results

### Participants

**Fig. 5:**
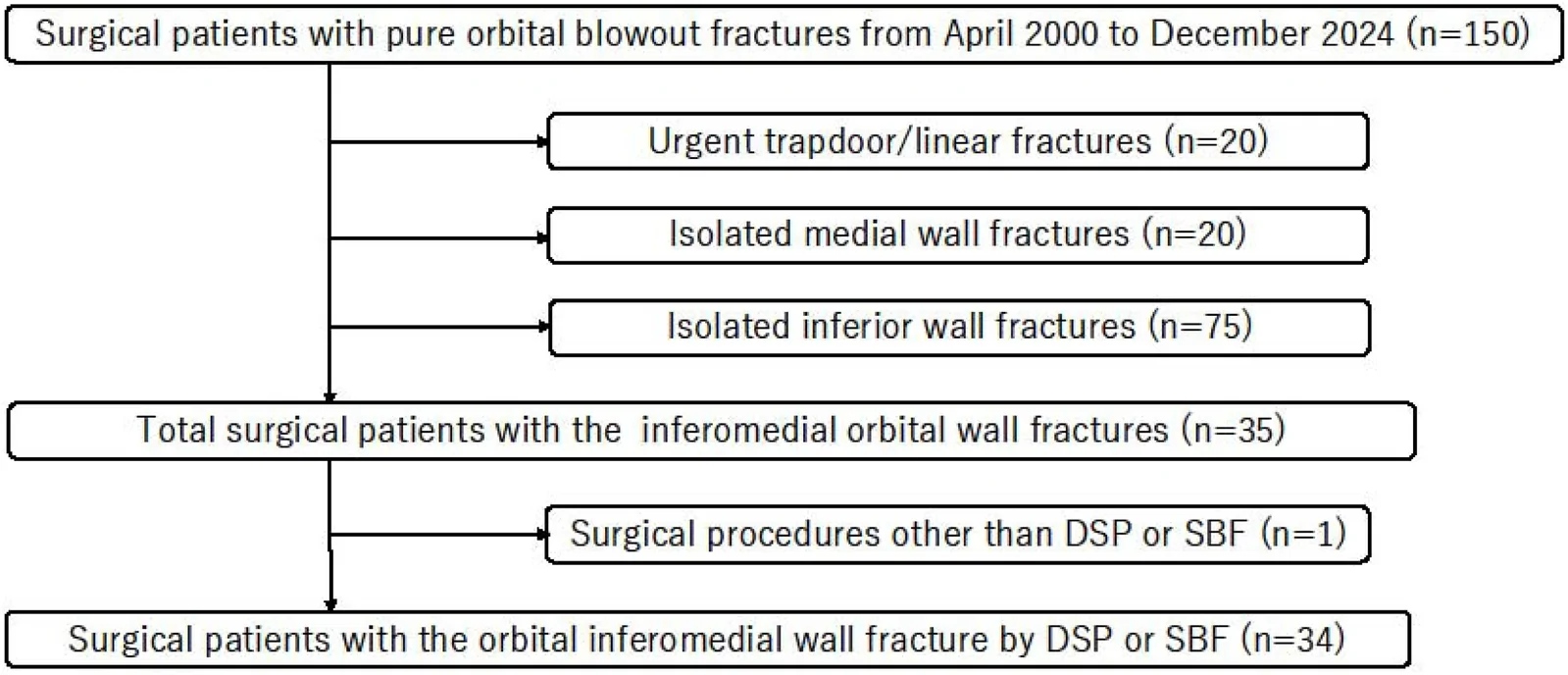
Flow diagram of patient selection and cohort derivation. Between April 2000 and December 2024, 150 patients underwent surgical treatment for pure orbital blowout fractures. After exclusion of urgent trapdoor or linear fractures (n = 20), isolated medial wall fractures (n = 20), and isolated inferior wall fractures (n = 75), 35 patients with inferomedial orbital wall fractures remained. One patient treated with a surgical procedure other than DSP or Silicone Balloon Fixation (SBF) was excluded, resulting in a final cohort of 34 consecutive patients treated with DSP or SBF.

**Fig. 6:**
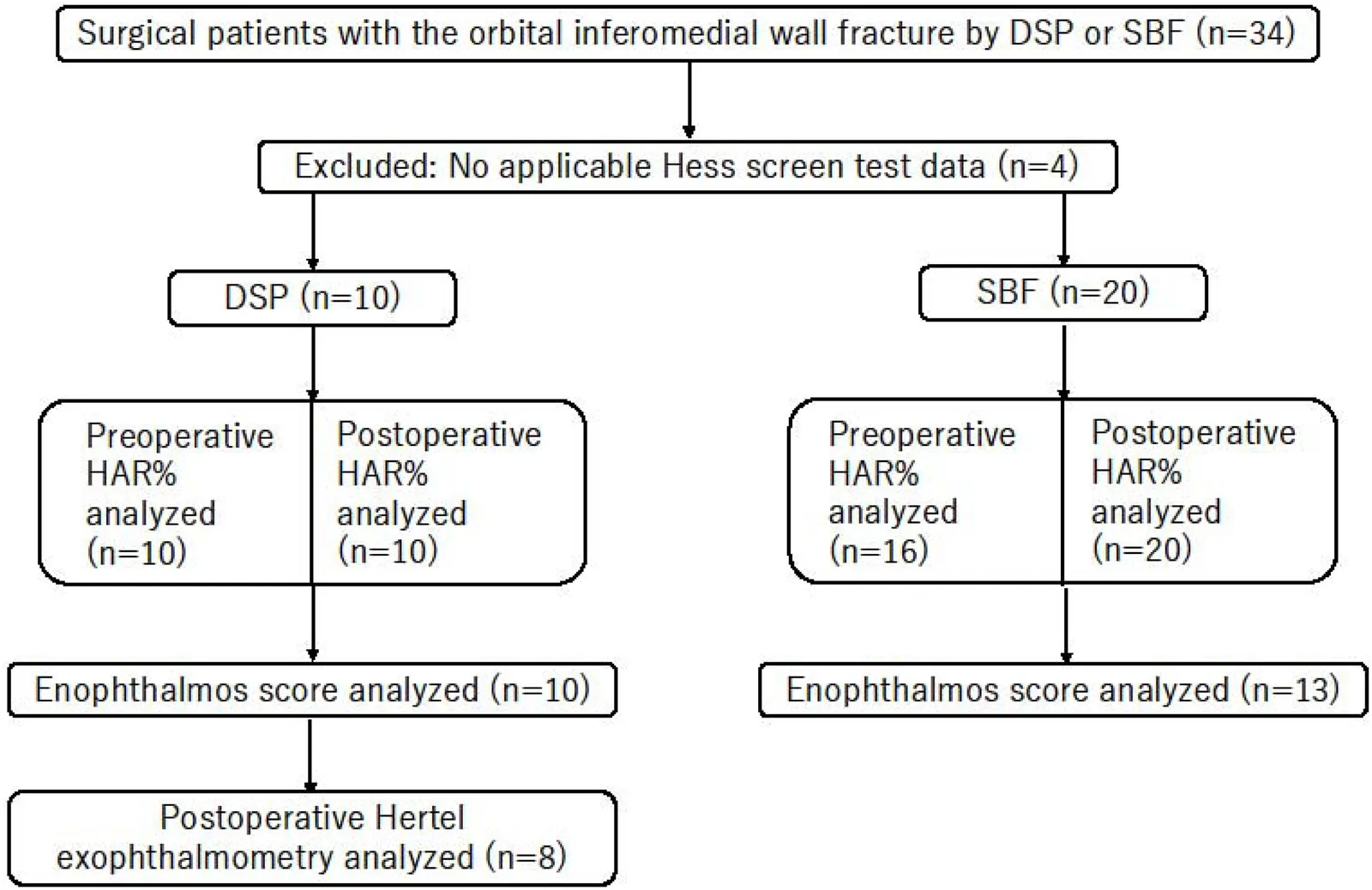
Flow diagram of the analysis cohort and availability of outcome data. From April 2000 to December 2024, 34 consecutive patients with inferomedial orbital wall fractures underwent surgical repair. Four patients were excluded owing to unavailable Hess screening data, leaving 30 patients for the analysis. The remaining cohort was divided into a DSP group (n = 10) and a SBF group (n = 20). Outcome datasets were available as follows: Hess area ratio (HAR%), preoperative (DSP, n = 10; SBF, n = 16) and postoperative (DSP, n = 10; SBF, n = 20); enophthalmos score (DSP, n = 10; SBF, n = 13); and postoperative Hertel exophthalmometry (DSP, n = 8).

The patient selection process is summarized in Fig. 5. This cohort represents the final set of eligible cases after screening according to the predefined criteria and does not reflect the total number of patients with pure orbital BOFs treated at our institution during the study period. The study participants are summarized in Fig. 6. A total of 34 cases were included, of which four were excluded owing to missing Hess screen test data. Consequently, 10 and 20 patients were enrolled in the DSP and SBF groups, respectively. The preoperative HAR%, postoperative HAR%, and enophthalmos scores were analyzed using an available case approach. The number of cases included in each analysis is shown in Fig. 6. Hertel exophthalmometry was performed in eight patients in the DSP group.

### Descriptive data (Table 1)

Baseline demographics were broadly comparable between the groups. The DSP cohort had a median age of 38.5-years, whereas the SBF cohort was 26.5-years (p = 0.15); men predominated in both groups (80% vs. 75% [p = 1.00]). The interval from injury to surgery was similar (median, 10-days [IQR 7–14] vs. 7-days [IQR 5–12] [p = 0.09]), as was postoperative length of stay (8-days [IQR 7.25–10.25] vs. 11-days [IQR 8.75–15.5] [p = 0.06]). Regarding follow-up, patients were observed for the primary outcome of enophthalmos for a median of 374-days (IQR 364–452) after DSP and 1965-days (IQR 978–3396) after SBF (p = 0.001). In contrast, follow-up for the secondary outcome of ocular motility was 374-days (IQR 364–452) in the DSP group and 102-days (IQR 62–291) in the SBF group (p = 0.001).

**Table 1:** Baseline characteristics of patients undergoing double-silicone procedure (DSP) and Silicone Balloon Fixation (SBF).

	DSP group	SBF group	
Characteristic	(n = 10)	(n = 20)	p
Male sex, n (%)^a^	8 / 10 (80 %)	15 / 20 (75 %)	1.00
Age, years^b^	38.5 (25.8–49.5)	26.5 (17.0–34.0)	0.15
Injury-to-surgery, days^b^	10 (7–14)	7 (5–12)	0.09
Postoperative length of stay^b^	8 (7.25–10.25)	11 (8.75–15.5)	0.06
Follow-up for enophthalmos, days^b^	374 (364–452)	1965 (978−3396)	0.001
Follow-up for ocular movement, days^b^	374 (364–452)	102 (62–291)	0.001

DSP, Double Silicone Procedure; SBF, Silicone Balloon Fixation.

Data are shown as median (IQR) for continuous variables or n/n (%) for categorical variables.

a Fisher’s exact test.

b Mann–Whitney *U*-test.

### Outcome data (Table 2)

#### Primary outcome – enophthalmos

Patients in the DSP group exhibited median enophthalmos score of 0 (IQR 0–0) versus 1 (IQR 0–1) in those in the SBF group (Hodges-Lehmann delta [HL Δ] -1.0, 95% CI -1.0–0.0 [p = 0.008]). In the DSP group, the postoperative enophthalmos difference was -1.00 mm (IQR -1.00–0.00 mm).

**Table 2:** Primary and secondary outcomes of DSP and SBF.

	DSP group	SBF group		
Outcome (nDSP/nSBF)	Median (IQR)	Median (IQR)	Hodges-Lehmann Δ (95 % CI)	p
Enophthalmos score (10 / 13)^b^	0 (0–0)	1 (0–1)	−1.0 (-1.0–0.0)	0.008
Postoperative enophthalmos difference^a^, mm (8/-)	−1.00 (-1.00–0.00)	‒	‒	‒
Preoperative HAR% (10 / 16)^b^	52.0 (26.0–70.2)	65.8 (41.9–76.5)	−9.5 (-21.3–0.5)	0.385
Postoperative HAR% (10 / 20)^b^	92.1 (86.0–100.0)	88.4 (80.4–97.0)	2.1 (0.0–7.8)	0.397

DSP, Double Silicone Procedure; SBF, Silicone Balloon Fixation.

a Mann–Whitney *U*-test.

b Negative values indicate regression from the healthy side; no Hertel exophthalmometry measurements were conducted in the SBF group.

#### Secondary outcomes – ocular motility

Preoperative HAR% was comparable between the DSP and SBF groups (52.0 [IQR 26.0–70.2] vs. 65.8 [IQR 41.9–76.5] [p = 0.385]), reflecting comparable motility restriction. Postoperative HAR% was comparable between the DSP and SBF groups (92.1 [IQR 86.0–100.0] vs. 88.4 [IQR 80.4–97.0], [p = 0.397]). The HL difference (DSP-SBF) in postoperative HAR% was +2.1 pp (95% CI 0.0 to 7.8). Because the lower CI bound (0.0 pp) exceeded the non-inferiority margin of –3 pp, DSP met the predefined criterion for non-inferiority (one-sided [p = 0.018], α = 0.025). Thus, DSP achieved an ocular motility recovery comparable and numerically superior to that of SBF.

### Adverse events

In the DSP and SBF groups, no iatrogenic injuries to the optic nerve or orbital contents were observed. No cases of infection or other complications related to silicone sheet placement were observed. Similarly, there were no instances of injury to the orbital contents or residual silicone material during silicone sheet removal. No clinically apparent eyelid malposition, such as entropion or ectropion, was observed during follow-up.

## Discussion

DSP demonstrated better outcomes in terms of postoperative enophthalmos compared to the conventional SBF. Furthermore, regarding POM, DSP yielded postoperative HAR% values comparable to SBF, thus fulfilling the prespecified non-inferiority margin of −3 pp; notably, the entire 95% CI lay above the diplopia-free threshold of HAR 85%, underscoring the clinical relevance of DSP.^19^ No adverse events were observed in patients treated with this surgical technique, and improved clinical outcomes were achieved compared to conventional methods.

In contemporary orbital reconstruction, rigid or semi-rigid implants ‒ such as titanium mesh and porous polyethylene, including preformed or patient-specific designs ‒ are widely used because they provide stable support and can improve reproducibility.^20^, ^21^, ^22^ However, recreating the complex three-dimensional internal orbital contour, particularly in extensive inferomedial defects, remains technically demanding, and implants requiring intraoperative shaping may be susceptible to fit and positioning errors.^21^^,^^22^ In addition, adverse events related to rigid reconstruction have been reported, including extraocular motility restriction caused by periorbital adhesion to titanium mesh and, rarely, vision-threatening compressive optic neuropathy.^23^^,^^24^ In contrast, silicone sheets are inexpensive and highly contourable, facilitating restoration of the inferomedial orbital curvature. Nevertheless, late complications after long-term silicone implantation such as cystic lesions, migration, infection, and implant exposure have also been described.^12^^,^^14^^,^^25^, ^26^, ^27^ Against this background, the DSP was designed to preserve the practical handling advantages of silicone while addressing concerns about prolonged implantation. By enabling transnasal silicone sheet removal as a brief outpatient otolaryngology procedure after healing, DSP avoids the need for dedicated removal surgery. Consequently, DSP minimizes the risk of long-term complications associated with silicone implants, allowing the surgeon to retain the benefits of silicone use without the associated drawbacks.^8^

Inferomedial fractures require reconstruction of a complex three-dimensional curved surface, and accurate restoration can be particularly challenging when the inferomedial orbital strut is compromised.^6^ Loss of the inferomedial strut and a large volume of herniated orbital contents increase the risk of postoperative enophthalmos and ocular motility disorders.^4^^,^^5^ Accordingly, surgical techniques that improve contour restoration and effective reduction of herniated contents may be advantageous. In the present cohort, DSP demonstrated superior outcomes in the prevention of postoperative enophthalmos, with no patients reporting subjective enophthalmos and no cases exceeding a 2 mm difference on Hertel exophthalmometry, a threshold commonly associated with clinically perceptible enophthalmos.^28^ Postoperative ocular motility remained within functionally acceptable limits.^19^ These findings indicate that DSP may be an effective surgical approach to minimize the risk of enophthalmos while preserving or improving ocular motility.

A combined transorbital-endoscopic approach has been reported for complex orbital wall fractures, as it provides an additional visualization corridor to the deep inferomedial orbit, enabling confirmation of fracture reduction and implant positioning while potentially limiting extensive orbital dissection.^17^^,^^29^ In particular, endoscopic assistance, sometimes with navigation, can aid intraoperative assessment of the posterior extent of defects and implant contouring in large inferomedial fractures, including those involving the inferomedial strut.^15^^,^^17^^,^^30^ In our study, we selected this combined approach for anatomically complex inferomedial fractures requiring broad yet controlled access. The multidirectional view was considered to support operative safety, and no clinically significant adverse events attributable to the endoscopic approach were observed. Although we used a subciliary approach in this series, the procedure is also feasible via a transconjunctival route; a transcaruncular route may further enhance medial exposure when required.

Various materials and techniques are used for orbital wall reconstruction, including titanium mesh, porous polyethylene, resorbable implants, autologous bone grafts, and patient-specific implants. Comparative syntheses suggest that patient-specific or pre-shaped approaches may improve accuracy and reduce operative time in some settings, although costs and resource utilization vary.^31^ In the present study, we focused on complex inferomedial defects and emphasized postoperative functional and cosmetic outcomes, as well as length of hospital stay. Evaluation of operative time and cost-effectiveness should be evaluated in future studies.

This study has some limitations. First, this was a single-center retrospective analysis with a small sample size (DSP, n = 10; SBF, n = 20), limiting generalizability and statistical precision. Second, enophthalmos was assessed using a standardized patient-reported scale, whereas objective Hertel exophthalmometry measurements were available only in the DSP cohort, introducing the potential for measurement bias. Third, follow-up schedules differed because ocular motility assessments were discontinued once stabilized, whereas enophthalmos was assessed at ≥6-months. Accordingly, outcomes were analyzed at clinically appropriate time points, although direct comparison of follow-up duration across measures is limited. Fourth, operative time and direct cost data were not collected. Finally, between-group comparisons should be interpreted as exploratory rather than confirmatory.

Despite these limitations, DSP appears to be a useful and safe option for the management of complex inferomedial orbital BOFs, with favorable functional and cosmetic outcomes. Larger prospective studies incorporating standardized objective assessments are warranted.

## Conclusions

In this single-center experience, DSP was associated with favorable postoperative outcomes, demonstrating improved enophthalmos control while preserving ocular motility. The use of easily handled, readily contoured silicone sheets supports procedural safety, and transnasal outpatient removal eliminates the need for an additional surgical procedure. DSP therefore represents a promising and practical option for reconstruction of complex inferomedial orbital BOFs.

## ORCID ID

Kosuke Takabayashi: 0000-0001-6519-1301

Yohei Maeda: 0000-0003-1510-9191

Nobuya Kataoka: 0000-0003-2942-1485

## Authors' contributions

KT analyzed the data and drafted the manuscript. YM analyzed the data. NK was responsible for the data collection. All authors made substantial contributions to the conception and design of the study and interpretation of the data. All authors have revised the manuscript, approved its submission for publication, and agreed to be accountable for all aspects of the work by ensuring that questions related to the accuracy or integrity of any part of the work are appropriately investigated and resolved.

## Ethical statement

Surgical treatment was performed after obtaining written informed consent from the patient. The study protocol was approved by the Ethics Committee of the Japanese Red Cross Asahikawa Hospital (approval number 201818-2).

## Funding

None.

## Data availability statement

The authors declare that all data are available in repository.

## Declaration of competing interest

The authors declare no conflicts of interest.
